# Personality Traits of Choral Singers and Their Association with Perceived Mental Well-Being

**DOI:** 10.3390/bs15050570

**Published:** 2025-04-23

**Authors:** Sibylle Robens, Alexandra Monstadt, Alexander Hagen, Thomas Ostermann

**Affiliations:** Department of Psychology and Psychotherapy, University Witten/Herdecke, 58455 Witten, Germany; alexandra.monstadt@uni-wh.de (A.M.); alexander.hagen@uni-wh.de (A.H.); thomas.ostermann@uni-wh.de (T.O.)

**Keywords:** choir singing, Big Five, personality traits, mental health, psychological well-being

## Abstract

Previous research indicates that choir singing enhances mental well-being. This study explores whether these well-being benefits are related to the personality traits of singers. We assessed the personality traits of 760 German amateur choral singers (205 men, 555 women, mean age 47.1 ± 14.0 years) using a 30-item version of the NEO–Five–Factor Inventory and compared them with a representative population sample. General mental well-being was measured with the WHO-5 well-being index, and perceived mental health benefits from singing were evaluated using the Bochum Change Questionnaire (BCQ-2000). Regression analyses examined the relationship between personality traits, BCQ-2000, and WHO-5 well-being scores. Choral singers scored significantly higher than the general population on extraversion, openness to experience, and agreeableness. The WHO-5 scores of choir singers were significantly positively correlated with extraversion and conscientiousness, and negatively with neuroticism. BCQ-2000 scores were significantly positively associated with extraversion, openness, and agreeableness. In this exploratory study, we examined the association between personality traits and singing-related mental well-being without accounting for other potential sociodemographic confounders of mental health, such as socioeconomic status. The study suggests that the self-reported mental well-being benefits of singing are influenced by individual personality traits.

## 1. Introduction

Choral singing is one of the most popular cultural activities in countries around the world. According to the European Choral Association (ECA, 2023), in Europe alone there are about 37 million choral singers. Singing in a choir requires a combination of psychosocial, physical, and cognitive performances that have been associated with health benefits in several studies. For example, investigations suggest that singing improves respiratory and cardiovascular functioning (Bernardi et al., 2017; Gick & Nicol, 2015; Lewis et al., 2016; Vickhoff et al., 2013), strengthens immune competence (Bernardi et al., 2017; Kreutz et al., 2005), stimulates cognitive functions (Mansens et al., 2018; Pentikäinen et al., 2021), and elevates mood (Kreutz et al., 2005; Unwin et al., 2002) and psychological well-being (Clift, 2012; Clift et al., 2010; Robens et al., 2022; Unwin et al., 2002).

The present study examines a sample of 760 German amateur choral singers, a subset of the 847 choristers analyzed by Robens et al. (2022). In their previous research, Robens et al. (2022) explored how sociodemographic factors, vocal characteristics, and choral participation influence the singing well-being of both amateur and professional singers. Their findings revealed benefits of singing across all age groups, genders, and education levels, suggesting that singing-related mental well-being can be enhanced through optimal singing conditions and dedicated participation in choral activities.

In contrast to singing individually, choral singers can experience a sense of cohesion, social support, and friendship within their choir, and it is believed that these factors also increase singing well-being (Clift et al., 2010). The impact of belonging to a social group on psychological well-being is supported by the investigation of Stewart and Lonsdale (2016). They compared self-reported psychological well-being in adult choral singers with those of solo singers or team sport players and found significantly higher well-being levels in choral singers and team sport players compared to solo singers.

Choral singing implies regular social contact and teamwork with other choir members. Compared to solo singers, choristers also have to coordinate and synchronize their actions, e.g., they have to blend their voices and have to adjust their volume within the group. At performances, choristers stand in front of an audience and possibly have to deal with stage fright. All these requirements suggest that emotionally stable and outgoing individuals might be more interested in a choir membership than introverted persons with high levels of neuroticism.

Reardon MacLellan (2011) compared Myers–Briggs personality types (Myers et al., 1998) among 355 students participating in a choir, orchestra, or band and found that choir members were more likely to be extraverted compared to orchestra students and that both groups were more likely to be intuitive and feeling compared to published norms for high school students. Sandgren (2019) found significantly higher Big Five levels of extraversion, agreeableness, and openness in 36 vocalists of a music college compared to a control group of 134 psychology students, whereas 72 instrumental students revealed no significant differences in personality traits compared to the control group. Torrance and Bugos (2017) used the Big Five IPIP (Goldberg, 1992) as a measure of personality traits in 137 college students and found high levels of openness in both vocalists and instrumentalists, but significantly higher levels of extraversion in vocalists compared to instrumentalists.

Other studies suggest that personality traits have an impact on psychological well-being. For example, Butkovic et al. (2012) found higher levels of emotional stability and extraversion to be associated with higher psychological well-being scores in 223 high school students and 134 older persons (54–90 years old). The results of Landa et al. (2010) also indicated that high scores in extraversion and low scores in neuroticism are associated with a higher psychological well-being. Grant et al. (2009) suggested, based on their study with 211 subjects, that in addition to extraversion and neuroticism, conscientiousness also predisposes to psychological well-being. Based on these study results, the reported positive effects of singing on mental well-being could possibly depend on the personality traits of people who are willing to attend a choir.

Choral singing is a self-selected activity that integrates musical expression, emotional involvement, and social interaction. Based on the studies cited above, we hypothesize that personality traits influence how individuals experience these aspects. An individual who is more extraverted may derive greater well-being benefits from group singing, as it involves active social engagement. In contrast, someone with higher levels of neuroticism may need additional support to feel comfortable and included, which could affect their mental well-being.

The purpose of this exploratory study was to identify the characteristic personality traits of choristers and to investigate whether these traits influence their reported mental well-being. It is important to note that the regression analysis conducted to examine the relationship between personality traits and choral singers’ mental well-being was adjusted for gender and age, but not for social and economic conditions, which are also important determinants of mental well-being (Kirkbride et al., 2024). We hypothesized that choir members tend to be more extroverted, open to experience, emotionally stable, and conscientious than subjects of the representative German sample of Körner et al. (2002). Furthermore, we hypothesized that the WHO-5 well-being index and reported positive changes in mental health from singing are negatively associated with neuroticism and positively associated with extraversion and conscientiousness in our sample.

## 2. Materials and Methods

### 2.1. Procedures

In a cross-sectional online survey, 847 adult choir members from Germany were recruited via email, Facebook, and two magazines published by the Choral Association of North Rhine-Westphalia and the German Choral Association. All respondents were active singers. To ensure a diverse sample, there were no restrictions on choir type or musical style.

Participants received an online consent form before starting the survey. The form assured them of the confidentiality of their responses and the voluntary nature of their participation. They could only begin the survey after providing informed consent. The study received approval from the Review Board of the Choral Association of North Rhine-Westphalia.

The questionnaire took approximately 20 min to complete and was administered using the LamaPoll online survey tool. The survey link was left open for five months. The survey included questions on socio-demographic and choir-related information, as well as questions on personality traits and perceived mental well-being. The sample was described in detail in Robens et al. (2022), where the authors focused on the influence of demographic characteristics and singing conditions rather than personality traits on singing-related psychological well-being.

### 2.2. Participants

For the present analysis, only adult participants (aged 18 and over) who were amateur singers and had been part of a choir for at least two years were included. Further, 760 of the 847 participants met these criteria, consisting of 555 women and 205 men, with an average age of *M* = 47.1 years (*SD* = 14.0).

A description of the demographics of the 760 participants is given in Table 1. The sample was characterized by a high level of education, with 78.5% holding a university or college degree. The majority of participants were employed or self-employed (70.4%) and 13.0% were retired. Most respondents had considerable experience of choral participation, with an average length of membership of 21 years (*SD* = 13.5). The majority (70.7%) did not sing solo parts in their choir. Different styles of choral music, such as sacred, secular, contemporary, and classical, were almost equally represented.

### 2.3. Intruments and Measurements

The participants filled out a series of questionnaires and part of the data are presented here. In addition to various questions about their socio-demographic background, their choir, and singing conditions, the participants answered questions about their Big Five personality traits, their current mental well-being, and their singing-related changes in mental health.

#### 2.3.1. NEO-FFI-30 Questionnaire

To assess personality traits, the study used the 30-item German version of the NEO–Five–Factor Inventory (NEO-FFI-30) questionnaire (Körner et al., 2002), which is based on Costa and McCrae’s Five-Factor Model (McCrae et al., 1989). This questionnaire includes six items for each of the Big Five personality dimensions: neuroticism, extraversion, openness to experience, agreeableness, and conscientiousness.

Participants rated each item on a five-point Likert scale, ranging from 0 (strongly disagree) to 4 (strongly agree), with a neutral midpoint of 2. After reverse coding nine of the items, the mean scores for each personality dimension were calculated. Higher scores reflect a stronger expression of the respective personality traits. Körner et al. (2008) reported good test–retest reliability and internal consistency for the NEO-FFI-30, with Cronbach’s α of 0.81 for neuroticism, 0.72 for extraversion, 0.67 for openness, 0.75 for agreeableness, and 0.78 for conscientiousness.

Reference values for the NEO-FFI-30 were obtained from a representative sample of 1908 German adults (aged 18 and older), comprising 1055 women (55.29%) and 853 men (44.71%), with a mean age of 47.68 years (*SD* = 16.92). This sample is described in detail by Körner et al. (2002), and the mean scores and standard deviations for the NEO-FFI-30 items were sourced from Körner et al. (2008).

#### 2.3.2. WHO-5 Well-Being Index

To evaluate participants’ mental well-being over the previous two weeks, the WHO-5 well-being index (Topp et al., 2015) was used. The index consists of five positively phrased statements: “I have felt cheerful and in good spirits”, “I have felt calm and relaxed,” “I have felt active and vigorous”, “I woke up feeling fresh and rested”, and “My daily life has been filled with things that interest me”. Each item is rated on a six-point Likert scale, ranging from 0 (at no time) to 5 (all of the time), with the total score calculated by summing the individual item ratings. A total score below 13 suggests poor well-being (Brähler et al., 2007). Several studies have demonstrated the high reliability and structural validity of the WHO-5 index across various countries and samples. Specifically, Brähler et al. (2007) reported a Cronbach’s alpha of 0.92 for the German version of the WHO-5 index.

#### 2.3.3. BCQ-2000 Questionnaire

Mental health changes resulting from choral singing were assessed using an adapted version of the Bochum Change Questionnaire (BCQ-2000) (Willutzki et al., 2013). Participants were asked to reflect on a time before they participated in a choir and compare it to their current experience as choir members, indicating their perceived mental health changes due to singing by agreeing or disagreeing with various statements.

The questionnaire included 26 bipolar items rated on a 7-point scale, ranging from 1 (totally disagree) to 7 (totally agree), with 4 representing a neutral value (no change). According to Willutzki et al. (2013), the BCQ-2000 has a high internal consistency, with Cronbach’s α = 0.96. The items are grouped into three subscales. After reverse scoring the negatively worded items, the average score for each subscale was calculated. Higher scores reflect positive mental health changes due to choral singing.

The first subscale, “Explicit Positive Change”, consists of 10 items describing improvements in feeling more relaxed, balanced, calm, and satisfied in daily life. The second subscale, “Reduction in Mental Stress”, includes 9 items related to greater self-acceptance, resilience, and perseverance, along with reduced feelings of being rushed, hopeless, or isolated. The third subscale, “Interaction Change”, involves 7 items assessing improvements in communication skills and the ability to remain confident and calm in social interactions.

### 2.4. Data Analysis

Statistical analyses were performed using IBM SPSS Statistics (Version 29.0, IBM Corp., Armonk, NY, USA) and *p*-values less than 0.05 were considered statistically significant. Demographic data, well-being scores, and Big Five dimensions were initially analyzed descriptively with means, standard deviations, or frequencies. Comparisons between the personality traits of the choir singers and those of a German population sample were analyzed using t-tests.

Differences in the personality traits of age and gender groups and of solo or non-solo singers were calculated using *t*-tests or F-tests with Scheffe’s post hoc tests. Cohen’s *d* and eta squared (*η*^2^) effect sizes were calculated for significant *t*- and F-test results to reveal the magnitude of the differences found. According to Cohen (1988), effect sizes were classified as small (0.2 ≤ *d* < 0.5; 0.01 ≤ *η*^2^ < 0.06), medium (0.5 ≤ *d* < 0.8; 0.06 ≤ *η*^2^ < 0.14), or large (d ≥ 0.8, *η*^2^ ≥ 0.14).

Bravais–Pearson correlation analyses and multiple regression models were conducted to investigate the strength of the relationship between the well-being scales and the personality traits. According to Cohen (1988), correlation coefficients were interpreted as small (0.1 ≤ |r| < 0.3), moderate (0.3 ≤ |r| < 0.5), or strong (|r| > 0.5).

## 3. Results

### 3.1. Reported Mental Well-Being

Table 2 presents descriptive statistics for the mental well-being variables. About one third of all participants (32.9%) rated their current psychological well-being as low (WHO-5 index < 13). On the other hand, according to the BCQ-2000 questionnaire, 85.6% stated that singing made them feel calmer and more emotionally balanced in everyday life, 87.0% reported a singing-induced reduction in mental stress, and 74.6% reported a positive change in interacting with others from singing.

### 3.2. Big Five Dimensions

Descriptive statistics of the personality traits of the total sample of the choir singers as well as the results separated by gender and age groups are shown in Table 3. The mean NEO-FFI-30 overall and in all subgroups were highest for Agreeableness and Conscientiousness, followed by Openness and Extraversion, and lowest for Neuroticism.

#### 3.2.1. Gender and Age Group Comparisons of Personal Traits

As shown in Table 3, women had higher mean values in all Big Five dimensions than men. Significant gender differences were found for neuroticism, *t*(758) = 3.87, *p* < 0.001, Cohen’s d = 0.32, agreeableness, *t*(758) = 3.49, *p* < 0.001, Cohen’s *d* = 0.29, and conscientiousness, *t*(758) = 2.66, *p* = 0.008, Cohen’s *d* = 0.22. There were no significant gender differences in the mean scores of extraversion and openness.

Regarding the four age groups, a main age effect was found for neuroticism, F(3, 756) = 22.21, *p* < 0.001, partial eta squared *η*^2^ = 0.08, with lower scores in individuals aged 60 and older compared to all other age groups (Scheffe’s post hoc tests, all *p*-values < 0.001). Concerning extraversion, openness, agreeableness, and consciousness, no significant age group main effects were found.

#### 3.2.2. Comparison of Choral Singers with Population Sample

**Figure 1:**
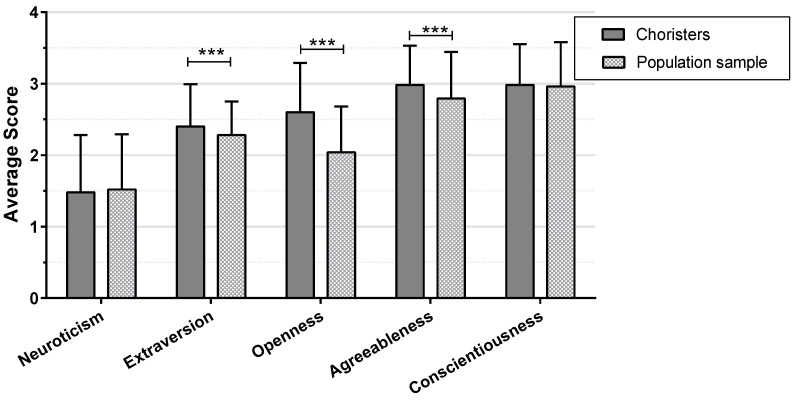
Means with standard deviations of choristers (N = 760) and reference population sample (N = 1908) for NEO-FFI-30. *t*-test results of pairwise comparisons between both samples, ***: *p* < 0.001.

In order to assess the magnitude of Big Five mean scores in the choristers’ sample, they were compared with the personality data from the reference German population sample of Körner et al. (2002). In Figure 1, mean scores and standard deviations of both samples are displayed.

Choristers showed significantly higher mean scores (*M* = 2.40, *SD* = 0.59) in extraversion than the subjects in the reference population (*M* = 2.28, *SD* = 0.47), *t*(2665) = 5.52, *p* < 0.001, Cohen’s *d* = 0.24. Singers also scored significantly higher in openness (*M* = 2.60, *SD* = 0.69) than the reference group (*M* = 2.04, *SD* = 0.64), *t*(2666) = 19.94, *p* < 0.001, Cohen’s *d* = 0.86. Furthermore, mean values of agreeableness were significantly higher in choir members (*M* = 2.98, *SD* = 0.55) compared to the reference group (*M* = 2.79, *SD* = 0.65), *t*(2666) = 7.11, *p* < 0.001, Cohen’s *d* = 0.30. The mean scores for neuroticism and conscientiousness did not differ significantly between the two samples.

### 3.3. Associations Between Personality Traits and Reported Mental Well-Being

Significant correlations were observed between the WHO-5 index and the three personality traits neuroticism (r = −0.49, *p* < 0.001), extraversion (r = 0.43, *p* < 0.001), and conscientiousness (r = 0.28, *p* < 0.001). Additionally, all three dimensions of the BCQ-2000 were positively associated with extraversion: Explicit Positive Change (r = 0.21, *p* < 0.001), Reduction in Mental Stress (r = 0.15, *p* < 0.001), and Interaction Change (r = 0.17, *p* < 0.001). There were also significant but small linear relationships between Explicit Positive Change and agreeableness (r = 0.15, *p* < 0.001), and between Reduction in Mental Stress and openness (r = 0.11, *p* = 0.002).

Regression models, adjusted for age and gender, were conducted with the WHO-5 well-being index or the BCQ-2000 dimensions as dependent variables and the Big Five personality traits as factors. The results are shown in Table 4.

The overall F-tests of all models were significant (*p* < 0.001), revealing a contribution of the personal traits on explaining singers’ well-being. The results showed a significant positive relationship between the WHO-5 well-being index and the traits of extraversion (*p* < 0.001) and conscientiousness (*p* = 0.003), while neuroticism was significantly negatively associated with the WHO-5 index (*p* < 0.001) (see Table 4). Additionally, there was a significant positive association between well-being and age (*p* = 0.010). Extraversion was significantly positively correlated with all three BCQ-2000 dimensions (see Table 4, all *p*-values ≤ 0.003). There was also a significant positive association between openness and Reduction in Mental Stress (*p* = 0.010), and agreeableness was positively associated with Explicit Positive Changes (*p* = 0.005).

## 4. Discussion

This study analyzed the Big Five personality traits (NEO-FFI-30) in 760 German amateur choral singers, revealing significant personality differences compared to a German population sample and examining how these traits are related to general mental well-being (WHO-5 index) and the perceived mental health benefits of singing (BCQ-2000).

Gender-based analyses revealed that male singers scored significantly lower than female singers on neuroticism, agreeableness, and conscientiousness, although the effect sizes were small. These results align with findings from Körner et al. (2002) and from other studies across different countries (Costa et al., 2001; Schmitt et al., 2017). Unlike Körner et al. (2002), who found lower scores for openness in men, our results suggest that male choral singers are as open to experience as female singers.

For neuroticism, there was a significant main effect of age with a large effect size. Singers aged 60 and older had significantly lower neuroticism scores compared to younger groups in our study. In contrast, Donnellan and Lucas (2008) stated a slight negative association of neuroticism with age in a large German Panel Study (N = 20,852) and Körner et al. (2002) found no association of age with neuroticism in their representative German population sample of 1908 subjects. This suggests that choral singing might enlarge emotional stability with age through providing a sense of community and social support.

The comparison of choral singers with the German population sample of Körner et al. (2002) confirmed our hypothesis that choral singers have significantly higher mean scores in extraversion and openness to experience. The effect size was small for extraversion and large for openness. Individuals with high levels in extraversion feel energized and excited in when around other people, and this characteristic is useful for singers in order to cooperate with other choir members and to handle the excitement of singing in front of an audience. Persons with high levels of openness are interested in new experiences and have a strong attachment to literature, art, and nature. An explanation for singers scoring high on this trait might be that singing is a cultural activity and choir members need to be open to new songs and arrangements.

The choral singers scored significantly higher on agreeableness than the general population sample. This difference may be explained by the fact that singing in a choir requires teamwork and good interpersonal relationships. There were no significant differences with the population sample in mean scores of neuroticism and conscientiousness.

Our findings align with prior studies; Sandgren (2019) observed significantly higher levels of extraversion, agreeableness, and openness in 36 singers compared to a control group of 134 students. The results of Reardon MacLellan (2011) indicated that students who sing in a choir were more likely to be extraverted compared to instrumentalists and high school norms. Cameron et al. (2015) found singers to be significantly more extroverted compared to bassists and more open than drummers.

Correlation and regression analyses supported the hypothesis that general mental well-being is positively associated with extraversion and conscientiousness and negatively associated with neuroticism. These findings are consistent with previous research (Anglim et al., 2020; Grant et al., 2009; Meléndez et al., 2019), which highlights these three traits as predictors of psychological well-being.

In the multiple regression analyses with changes in mental health from singing (BCQ-2000 scores) as the dependent variable, the dimensions of extraversion, agreeableness, and openness were identified as relevant factors. The results suggest a positive association of these three personality traits with the perceived mental health benefits. Extraversion was significantly positively associated with all three BCQ-2000 scores, openness was positively related to a reduction in mental stress, and agreeableness was positively associated with being more relaxed, calm, and satisfied in daily life.

The observed significant correlation coefficients indicate small-to-modest relationships. Although significant, these associations suggest that personality traits are only one of many factors contributing to singing-related mental well-being. This is supported by Robens et al. (2022), who have identified engagement in choral activities and optimal singing conditions, such as vocal warm-up and singing in a comfortable vocal range, as relevant factors. Another important factor associated with general mental well-being, according to recent studies, is subjective socioeconomic status (SES) (e.g., Kamalulil & Panatik, 2021; Navarro-Carrillo et al., 2020). The SES might also be connected with the reported well-being of choral singers, as people with higher SES might be more likely to sing in choirs. Besides age and gender, we did not control for other potential sociodemographic confounders of singers’ mental well-being in this explanatory study.

A limitation of this study is that it is an exploratory analysis of cross-sectional data. To test for the existence of cause–effect relationships, a longitudinal design would be more appropriate. Additionally, further longitudinal research could clarify how aspects of choir involvement (e.g., social cohesion, shared identity, and synchrony) improve mental health, especially among individuals predisposed to benefits (e.g., extraverts).

When interpreting the results, it must be taken into account that the proportion of women in the choir sample was higher than in the reference sample. Additionally, the reference sample was from 2002, and its demographic distribution (e.g., age and gender) is not representative of the current German adult population. However, to our knowledge, it is the only large population sample in which participants completed the German version of the NEO-FFI-30. This enables us to compare our mean Big-Five values with theirs. Moreover, personality traits are generally considered to be relatively stable across different situations and over time (Harris et al., 2016).

Although this exploratory study did not control for social determinants of mental health, the findings suggest that personality traits play a meaningful role in how individuals experience the benefits of choral singing. Based on our findings, the effects of singing on well-being reported in several choir studies (Clift, 2012; Clift et al., 2010; Robens et al., 2022; Unwin et al., 2002) may have been influenced by the fact that choir members tend to be more extroverted, agreeable, and open to experience than the general population.

## Figures and Tables

**Table 1:** Numbers with frequencies or means and standard deviations of demographic data of the chorister sample.

	*n*	% or *M* (*SD*)
Gender		
Male (m)	205	27.0%
Female (f)	555	73.0%
Age (years)	760	47.10 (14.03)
18–29	114	15.0%
30–44	181	23.8%
45–59	327	43.0%
≥60	138	18.2%
Graduation ^1^		
High	596	78.5%
Intermediate	131	17.3%
Basic	26	3.4%
Other	6	0.8%
Employement Status		
Student	93	12.2%
Employed	442	58.2%
Self-employed	93	12.2%
Homemaker	15	2.0%
Retired	99	13.0%
Unemployed	9	1.2%
Other	9	1.2%
Martial status		
Single	159	20.9%
Married/in a relationship	302	39.7%
Divorced	28	3.7%
Widowed	11	1.4%
N/A	7	0.9%
Years singing in choir(s)	760	21.12 (13.49)
Singing solo parts		
yes	223	29.3
no	537	70.7
Choral music style (multiple answers)		
Sacred	492	64.7
Secular	447	58.8
Contemporally	410	53.9
Classical	470	61.8

Note. ^1^ Education level: High = German Abitur (university-entrance diploma) or Fachabitur (technical diploma); Intermediate = German Realschulabschluss (general certificate of secondary education); Low = German Hauptschulabschluss (general school certificate).

**Table 2:** Numbers with frequencies or means and standard deviations of well-being scores of the chorister sample (N = 760).

	*n*	% or *M* (*SD*)
WHO-5 general well-being	760	14.61 (4.85)
Low (<13)	250	32.9%
Moderate to good (≥13)	510	67.1%
Mental health changes from singing (BCQ-2000)		
● Explicit positive change	758	4.98 (0.88)
Negative or neutral (≤4)	106	13.9%
Positive (>4)	652	85.8%
● Reduction in mental stress	758	4.94 (0.88)
Negative or neutral (≤4)	97	12.8%
Positive (>4)	661	87.0%
● Interaction change	758	4.74 (0.83)
Negative or neutral (≤4)	191	25.1%
Positive (>4)	567	74.6%

**Table 3:** Means and standard deviations of the choristers’ personality traits grouped by gender and age.

		NEO-FFI-30 Big Five Personality Traits				
		Neuroticism	Extraversion ^1^	Openness to Experience	Agreeable-Ness	Conscientious- Ness
	*n*	*M* (*SD*)	*M* (*SD*)	*M* (*SD*)	*M* (*SD*)	*M* (*SD*)
Total	760	1.48 (0.80)	2.40 (0.59)	2.60 (0.69)	2.98 (0.55)	2.98 (0.57)
Gender						
Male	205	1.30 (0.81)	2.37 (0.58)	2.56 (0.70)	2.87 (0.59)	2.89 (0.59)
Female	555	1.55 (0.79)	2.41 (0.60)	2.61 (0.69)	3.03 (0.52)	3.01 (0.56)
Age (yrs)						
18–29 yrs	114	1.76 (0.83)	2.49 (0.61)	2.54 (0.76)	3.00 (0.61)	2.93 (0.61)
30–44 yrs	181	1.68 (0.80)	2.34 (0.58)	2.52 (0.77)	2.90 (0.53)	2.90 (0.53)
45–59 yrs	327	1.45 (0.77)	2.37 (0.60)	2.64 (0.67)	2.99 (0.53)	2.99 (0.53)
≥60 yrs	138	1.07 (0.64)	2.46 (0.57)	2.64 (0.57)	3.06 (0.54)	3.09 (0.58)

Note. ^1^ One female, age 22 years, has a missing value for extraversion.

**Table 4:** Multiple regression analyses with WHO-5 Index and BCQ-2000 scales as dependent variables, and age, gender, and Big Five dimensions as factors.

	Dependent Variable																							
	WHO-5 Well-Being Index						BCQ-2000 Explicit Positive Change						BCQ-2000 Reduction in Mental Stress						BCQ-2000 Interaction Change					
	b	*SE*(b)	β	95% CI(b)		*p*	b	*SE*(b)	β	95% CI(b)		*p*	b	*SE*(b)	β	95% CI(b)		*p*	b	*SE*(b)	β	95% CI(b)		*p*
				*LL*	*UL*					*LL*	*UL*					*LL*	*UL*					*LL*	*UL*	
Intercept	7.56	1.61		4.40	10.72	<0.001	3.75	0.35		3.07	4.42	<0.001	4.06	0.35		3.37	4.75	<0.001	4.17	0.33		3.53	4.82	<0.001
Age (y)	0.03	0.01	0.08	0.01	0.05	0.010	−0.03	0.02	−0.05	−0.07	0.02	0.227	−0.04	0.02	−0.06	−0.01	0.01	0.134	−0.04	0.02	−0.06	−0.01	0.01	0.099
Gender (Ref. male)	−0.01	0.34	−0.001	−0.68	0.66	0.968	−0.07	0.07	−0.04	−0.22	0.07	0.319	−0.01	0.08	−0.01	−0.16	0.13	0.847	−0.18	0.07	−0.10	−0.32	−0.04	0.010
Neuroticism	−1.95	0.22	−0.32	−2.38	−1.51	<0.001	−0.02	0.05	−0.02	−0.11	0.08	0.717	−0.03	0.05	−0.02	−0.12	0.07	0.595	0.01	0.05	0.01	−0.08	0.10	0.771
Extraversion	2.40	0.27	0.29	1.87	2.93	<0.001	0.25	0.06	0.17	0.13	0.36	<0.01	0.16	0.06	0.11	0.05	0.28	0.06	0.22	0.06	0.15	0.11	0.33	<0.01
Openness	0.05	0.21	0.01	−0.37	0.47	0.803	0.06	0.05	0.05	−0.03	0.15	0.161	0.12	0.05	0.10	0.03	0.21	0.010	0.08	0.04	0.07	−0.01	0.16	0.074
Agreeableness	0.08	0.28	0.01	−0.48	0.64	0.771	0.17	0.06	0.12	0.05	0.29	0.05	0.05	0.06	0.03	−0.07	0.17	0.446	0.10	0.06	0.07	−0.01	0.21	0.085
Conscientiousness	0.84	0.28	0.10	0.29	1.39	0.003	0.08	0.06	0.05	−0.03	0.20	0.161	0.09	0.06	0.06	−0.03	0.21	0.138	0.0	0.06	0.03	−0.11	0.12	0.945
Model Fit	*F*(7, 750) = 52.11 *** R^2^ (adj. *R*^2^) = 0.327 (0.321)						*F*(7, 750) = 7.271 *** *R*^2^ (adj. *R*^2^) = 0.064 (0.055)						*F*(7, 750) = 4.164 *** R^2^ (adj. *R*^2^) = 0.037 (0.028)						*F*(7, 750) = 5.077 *** *R*^2^ (adj. *R*^2^) = 0.045 (0.036)					

Note. *n* = 758 included in the regression analyses, b = unstandardized regression coefficient, β = standardized regression coefficient, *SE*(b) = standard error of b, CI(b) = confidence interval of b, *LL* = lower limit, *UL* = upper limit, ***: *p* < 0.001.

## Data Availability

The data that support the findings of this study are available from the corresponding author, [S.R.], upon reasonable request.
